# Impact of chemotherapy and/or immunotherapy on neutralizing antibody response to SARS‐CoV‐2 mRNA‐1237 vaccine in patients with solid tumors

**DOI:** 10.1002/1878-0261.13359

**Published:** 2022-12-30

**Authors:** Eudald Felip, Edwards Pradenas, Margarita Romeo, Silvia Marfil, Benjamin Trinité, Víctor Urrea, Ainhoa Hernández, Ester Ballana, Marc Cucurull, Lourdes Mateu, Marta Massanella, Bonaventura Clotet, Teresa Morán, Julià Blanco

**Affiliations:** ^1^ IrsiCaixa AIDS Research Institute Badalona Spain; ^2^ Medical Oncology Department, Catalan Institute of Oncology – Badalona Badalona Applied Research Group in Oncology (B‐ARGO) Spain; ^3^ Germans Trias i Pujol Research Institute (IGTP) Badalona Spain; ^4^ CIBER Infectious Diseases (CIBERINFEC), Carlos III Institute of Health (ISCIII) Madrid Spain; ^5^ Infectious Diseases Department Hospital Universitari Germans Trias i Pujol Badalona Spain; ^6^ Fundació Lluita contra les Infeccions Hospital Universitari Germans Trias i Pujol Badalona Spain; ^7^ Centro de Investigación Biomédica en Red de Enfermedades Respiratorias (CIBERES), Carlos III Institute of Health (ISCIII) Madrid Spain; ^8^ University of Vic–Central University of Catalonia (UVic‐UCC) Spain

**Keywords:** adverse reactions, anticancer therapy, cancer, COVID‐19, moderna vaccine, neutralizing antibodies

## Abstract

Patients with solid tumors have been a risk group since the beginning of the SARS‐CoV‐2 pandemic due to more significant complications, hospitalizations or deaths. The immunosuppressive state of cancer treatments or the tumor itself could influence the development of post‐vaccination antibodies. This study prospectively analyzed 89 patients under chemotherapy and/or immunotherapy, who received two doses of the mRNA‐1237 vaccine, and were compared with a group of 26 non‐cancer individuals. Information on adverse events and neutralizing antibodies against the ancestral strain of SARS‐CoV‐2 (WH1) have been analyzed. Local reactions accounted for 65%, while systemic reactions accounted for 46% of oncologic individuals/cancer patients. Regarding the response to vaccination, 6.7% of cancer patients developed low neutralizing antibody levels. Lower levels of neutralizing antibodies between cancer and non‐cancer groups were significant in individuals without previous SARS‐CoV‐2 infection, but not in previously infected individuals. We also observed that patients receiving chemotherapy or chemoimmunotherapy have significantly lower levels of neutralizing antibodies than non‐cancer individuals. In conclusion, our study confirms the importance of prioritizing cancer patients receiving anticancer treatment in SARS‐CoV‐2 vaccination programs.

## Graphical abstract

Cancer patients are a risk group for SARS‐CoV‐2 infection. We determined the levels of anti‐SARS‐CoV‐2 plasma neutralizing antibodies post mRNA‐1273 vaccination and observed that cancer patients exhibit lower titers compared to non‐cancer individuals, particularly in individuals not previously infected by SARS‐CoV‐2. Additionally, we found an association between low levels of neutralizing antibodies and chemotherapy‐containing treatments, highlighting their prioritization in SARS‐CoV‐2 vaccination programs.

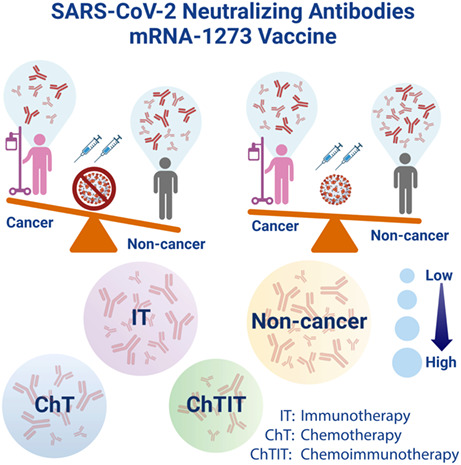

**Abbreviations**
AE: adverse events
ChTIT: chemoimmunotherapy
ChT: chemotherapy
COVID‐19: Coronavirus disease 2019
FDR: false discovery rate
GMT: geometric mean titer
ID_50_
: half‐maximal inhibitory dilution
IT: immunotherapy
IQR: interquartile range
NLR: neutrophil : lymphocyte ratio
PCR: polymerase chain reaction
SARS‐CoV‐2: severe acute respiratory syndrome coronavirus 2
VSV: vesicular stomatitis virus
WH1: Wuhan‐Hu‐1

## 1 Introduction

Since the beginning of the SARS‐CoV‐2 pandemic, cancer patients were considered a risk group in case of infection [1]. According to initial data from the Chinese population during the SARS‐CoV‐2 surge in 2019, the likelihood of mortality was estimated at 6.2 times higher when cancer patients with early‐stage and healthy individuals were compared [5.6 vs. 0.9] [2]. Higher infection and mortality rates in cancer patients could be explained by their immunosuppressed status due to both the tumor and the anticancer treatments [3]. In addition, frequent attendance at healthcare facilities may increase the risk of contracting the infection. However, other studies did not find an additional risk of SARS‐CoV‐2 infection‐related mortality when cancer patients were compared to the general population [4].

Some studies have focused on validating the prognostic value of several factors and parameters in SARS‐CoV‐2 infected patients with cancer, such as systemic inflammation (measured by the neutrophil : lymphocyte ratio [NLR]), lymphocytopenia or hypoalbuminemia to identify patients at risk of severe disease [5]. Consequently, a better understanding of COVID‐19, an earlier diagnosis, and improved management [6] led to decreased mortality in cancer patients with SARS‐CoV‐2 infection in Europe [7]. Nevertheless, despite a reduction in mortality, SARS‐CoV‐2 infection has impacted cancer patients due to delayed diagnosis and treatment initiation or major hospitalization and complications [8]. Moreover, patients with cancer may have been less likely to be accepted in Intensive Care Units, less likely to have their care escalated, and more likely to die when admitted with COVID‐19 [9]. To reduce the impact of the infection, widespread vaccination programs have been deployed. Initially, risk groups, including cancer patients, especially those in active cancer treatment, were prioritized in most countries, including Spain.

In the Catalan Institute of Oncology (Spain), the mRNA‐1273 vaccine was offered urgently to all cancer patients under treatment. The mRNA‐1273 vaccine, developed by Moderna®, is a lipid nanoparticle‐encapsulated mRNA‐based vaccine encoding the spike protein of the ancestral SARS‐CoV‐2 and is administered in two doses at a 28‐day interval. The phase 3 trial enrolled 30 420 volunteers and showed that symptomatic COVID‐19 illness was reduced drastically in the vaccine group (11 vs. 185 participants) with an efficacy of 94.1%. No safety concerns were identified other than transient local and systemic reactions [10]. However, since pivotal vaccine trials did not include cancer patients under cancer treatment, there was a lack of evidence of vaccine safety and efficacy during cancer treatment. Mirroring other viral infections, such as influenza, low immunogenicity against SARS‐CoV‐2 or after SARS‐CoV‐2 vaccines might be foreseen [11, 12], turning cancer patients a priority for vaccination.

Some adverse events (AE) after vaccination have been reported, mostly graded as mild, however rare, but life‐threatening AE such as myocarditis were also noted in patients with cancer [13, 14]. In general, the concomitant administration of chemotherapy (ChT) or immunotherapy (IT) has not demonstrated an increase in toxicity [15, 16]. In this prospective study, we analyzed the neutralizing activity and safety of the mRNA‐1273 vaccine in a cohort of patients with solid tumors undergoing different active anticancer treatments (ChT, IT, or chemoimmunotherapy [ChTIT]) during vaccination compared to a non‐cancer control group.

## 2 Material and methods

### 2.1 Participants

This cohort study included patients with solid tumors receiving cancer treatment, including ChT, IT or ChTIT (Table S1). All patients received treatment in the Catalan Institute of Oncology (ICO)‐Badalona Medical Oncology Department at the Hospital Germans Trias i Pujol, in Spain. ICO hosted a massive vaccination point and all patients receiving active treatment were called to receive their vaccines. This warning for vaccination was independent of prior SARS‐CoV‐2 infection. All patients received two doses of the mRNA‐1273 vaccine, 28 days apart. Blood samples were collected before the first and second doses and 2–3 weeks after the second one.

The study investigators accessed electronic health records to review patients' clinical data [cancer type, concomitant medication, treatments dates, comorbidities, previous SARS‐CoV‐2 infection by positive polymerase chain reaction (PCR), and demographic characteristics] and laboratory test results (complete blood cell count). All patients were followed up within at least, the first month after full vaccination to record local and systemic reactions related to the vaccine. The study methodologies conformed to the standards set by the Declaration of Helsinki, and the ethical committee approved the study at the Hospital Germans Trias i Pujol (SERCOVID? PI‐20‐202). All participating patients signed the informed consent; no financial compensation was provided.

For comparative purposes, a group of 26 healthy individuals having received two doses of the mRNA‐1273 vaccine (from the KING cohort extension of the Hospital Germans Trias i Pujol, PI‐20‐217) was selected. Samples from this control group were obtained 15 [IQR: 14–16] days after the second dose of the vaccine.

### 2.2 Pseudovirus generation and neutralization assay

HIV reporter pseudoviruses expressing the Wuhan‐Hu‐1 (WH1) spike protein and luciferase were produced as previously described [17, 18]. Neutralization assays were performed in duplicate. Briefly, 200 TCID_50_ of pseudovirus were preincubated with three‐fold serial dilutions (1/60–1/14 580), of heat‐inactivated plasma samples, for 1 h at 37 °C. Then, 1 × 10^4^ HEK293T/hACE2 cells treated with DEAE‐Dextran (Sigma‐Aldrich, Madrid, Spain) were added. Results were read after 48 h using the EnSight Multimode Plate Reader and BriteLite Plus Luciferase reagent (PerkinElmer, Madrid, Spain). The values were normalized and reported as a 50% neutralization titer [reciprocal half‐maximal inhibitory dilution (ID_50_)] based on a four‐parameter regression curve in prism 9 (GraphPad Software, San Diego, CA, USA). This assay has been previously validated with a replicative viral inhibition assay [19] and VSV pseudoviruses were used as a specificity control.

### 2.3 Statistical analysis

Continuous variables were described using medians and the interquartile range (IQR), whereas categorical variables were reported as percentages over available data. The different groups were compared using Pearson's Chi‐squared test, Fisher's exact test, Kruskal–Wallis rank‐sum test, and Nonparametric left‐censored multicomparison test (based on Peto–Peto rank test) with Benjamini–Hochberg's FDR correction. Two‐tailed *P*‐value ≤ 0.05 was considered statistically significant. All analyses were performed using graphpad prism 9.1.0 (GraphPad Software) and r 3.6.3 (R Foundation for Statistical Computing).

## 3 Results

### 3.1 Clinical data

This study included 89 patients with solid tumors (median 65 years old and 44.9% female) and 26 individuals without cancer as a control group (median 40 years old and 65% female). The most frequent tumors in the cancer cohort were non‐small cell lung cancer (56.2%) and breast cancer (19.1%). All cancer patients were receiving active treatment: 41 patients in the ChT group (mainly platinum‐based therapy, taxanes and capecitabine), 34 patients in the IT group (mostly on pembrolizumab and nivolumab) and 14 patients in the ChTIT group (> 70% on pembrolizumab, Table S1). 76.4% of patients had an advanced tumor. SARS‐CoV‐2 infection before vaccination was recorded in 24.7% of the patients, all of whom had a mild or asymptomatic disease (Table 1). The most frequent comorbidities included hypertension (41.6%) and a history of chronic obstructive pulmonary disease (37.1%). Seventy‐three percent of the patients were current or former smokers. No statistical differences in comorbidities nor in use of daily oral corticosteroids were found between the three treatment groups. As expected, a higher value of leukocytes and hemoglobin was found in the IT group alone compared to ChT‐containing regimens (7.6 vs. 5.8 and 6.2, *P* = 0.018 for leukocytes; 13.2 vs. 11.7 and 11.9, *P* < 0.001 for hemoglobin in IT, ChT and ChTIT groups, respectively) at baseline. No patient exhibited neutropenia before any dose of vaccine. At the time of the analysis, 62.9% of patients were still alive (Table S2).

**Table 1:** Patient's cohort characteristics. ChT, chemotherapy; ChTIT, chemoimmunotherapy; ECOG, Eastern cooperative oncology group; IT, immunotherapy; *n*, number of patients; NSCLC, non‐small cell lung cancer; PS, performance status; SCLC, small cell lung cancer.

Characteristic	ChT, *n* = 41	IT, *n* = 34	ChTIT, *n* = 14	*P*‐value^a^
Gender, *n* (%)				
Male	17 (41%)	24 (71%)	8 (57%)	0.042
Female	24 (59%)	10 (29%)	6 (43%)	
Age (years), median [IQR]	63 [55–74]	65 [57–70]	66 [63–72]	0.529
Previous SARS‐CoV‐2 infection, *n* (%)	10 (24%)	9 (26%)	3 (21%)	>0.999
PS‐ECOG, *n* (%)				
0	12 (29%)	13 (38%)	5 (36%)	0.657
1	24 (59%)	20 (59%)	8 (57%)	
2	5 (12%)	1 (2.9%)	1 (7.1%)	
Primary tumor, *n* (%)				
Breast	17 (41%)	0 (0%)	0 (0%)	<0.001
NSCLC	9 (22%)	29 (85%)	12 (86%)	
SCLC	3 (14.4%)	2 (5.9%)	0 (0%)	
Others^b^	3 (8.8%)	9 (22%)	2 (14%)	
Cancer stage, *n* (%)				
Local	12 (29%)	8 (24%)	1 (7.1%)	0.247
Advanced	29 (71%)	26 (76%)	13 (93%)	

^a^ Pearson's Chi‐squared test; Fisher's exact test; Kruskal–Wallis rank sum test.

^b^ Include: Gynecologic, colorectal, melanoma, pancreatic and sarcoma tumors.

### 3.2 Adverse events related to mRNA‐1273 vaccine

**Fig. 1:**
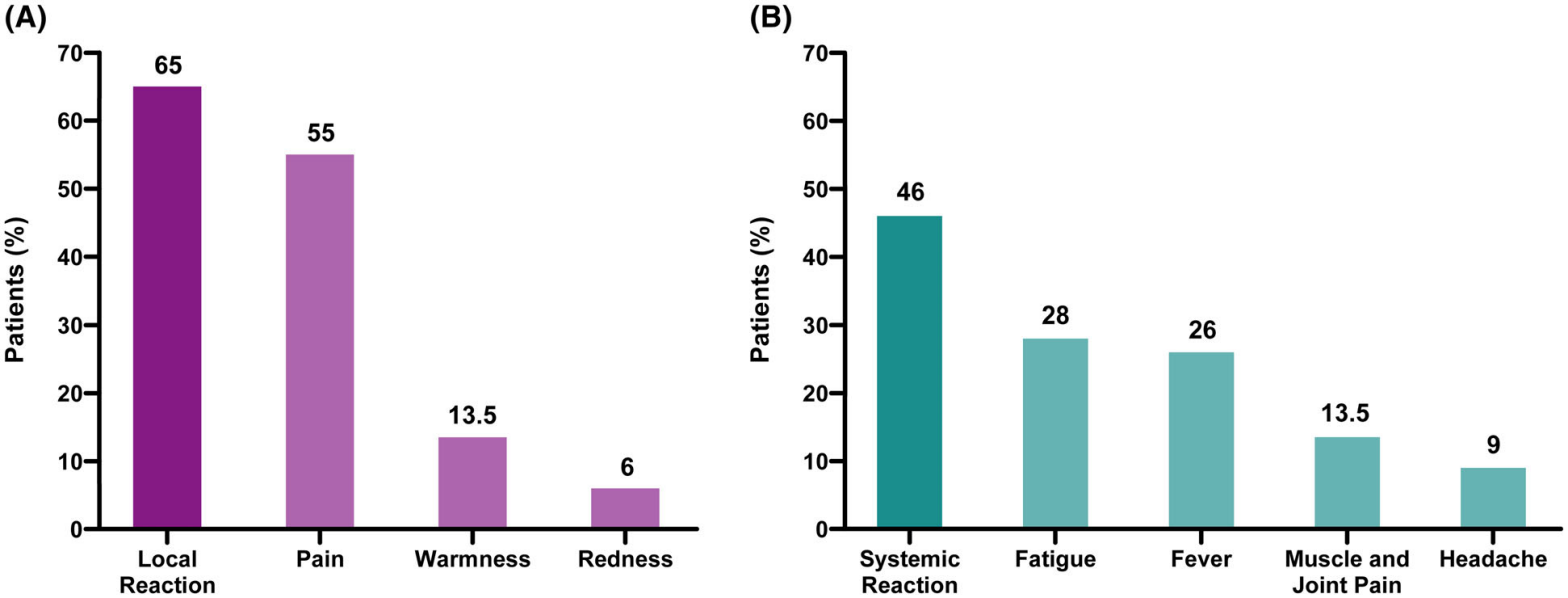
Side effects related to mRNA‐1237 vaccine in solid tumor cohort receiving active cancer treatment (*n* = 89). Column bar graphs show the frequency of (A) total local reactions (65%) and main different local reactions, and (B) total systemic reactions (46%) and main different systemic reactions.

One month after complete vaccination, oncology patients were questioned about reactions to any of the two doses. The effects of the first and second doses were jointly reported. Overall, 65% of patients reported local reactions, with pain at the injection site being the most frequent (55%). A lower percentage of patients reported other injection side effects, such as warmness (13.5%) and redness (6%, Fig. 1A). Most of the local reactions lasted less than 96 h. In addition, 46% of the patients reported some systemic reaction, being fatigue (28%), fever (26%), muscle and joint pain (13.5%) and headache (9%) the most common ones (Fig. 1B). No differences were found in adverse effects among the different treatment groups or according to previous SARS‐CoV‐2 infection (Tables S3 and S4). No hospitalizations due to vaccine complications were reported. 46% of the patients took symptomatic treatment (acetaminophen or ibuprofen). There were no delays in the administration of the anticancer therapy due to vaccine‐associated AE.

### 3.3 The status of previous SARS‐CoV‐2 infection determines neutralizing activity after vaccination

**Fig. 2:**
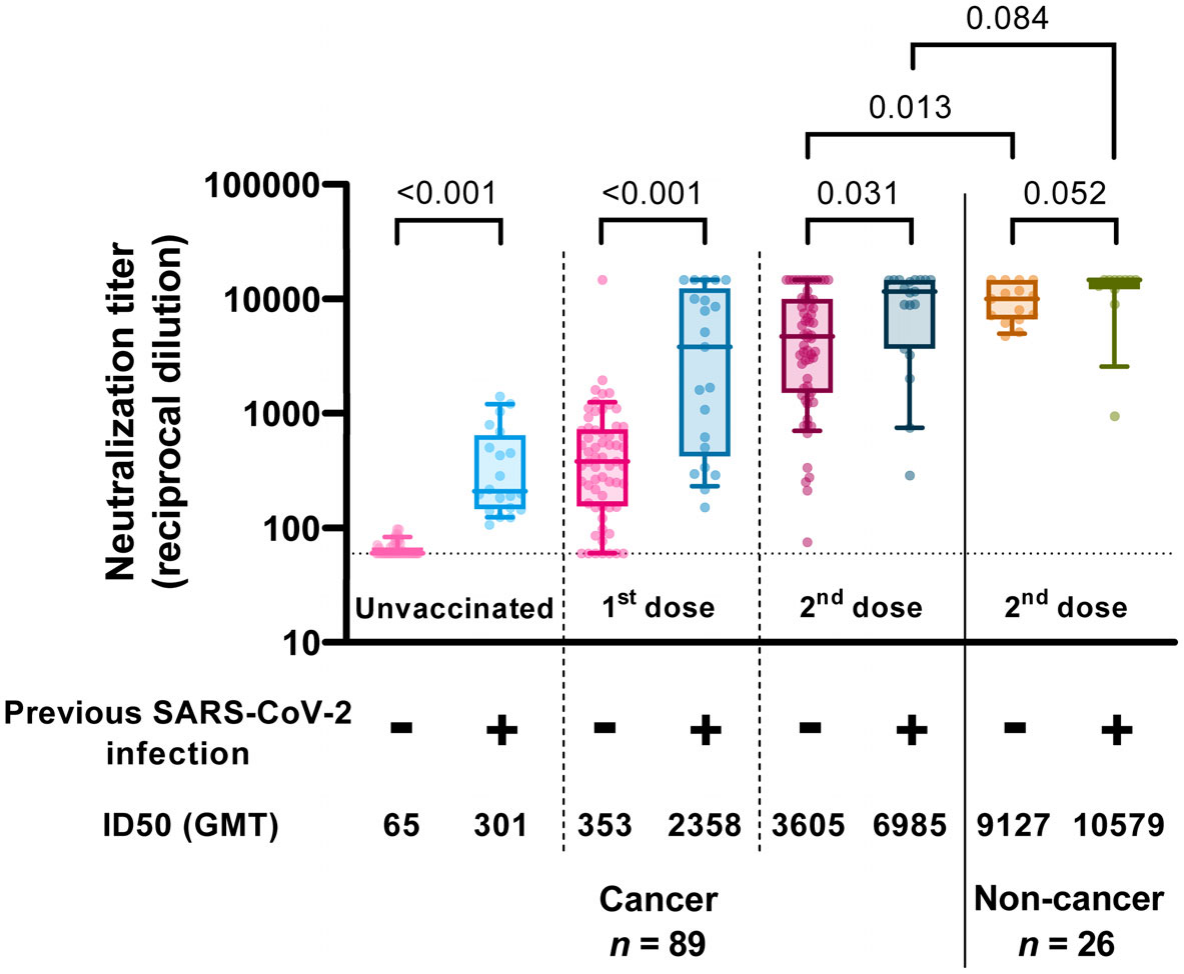
Neutralizing activity against WH1. Neutralization titers from participants' plasmas at different time points. Boxes show the median and the interquartile range (25^th^ and 75^th^ percentiles) and whiskers the 10^th^ and 90^th^ percentiles. *P* values correspond to Peto–Peto rank test with Benjamini & Hochberg adjustment. Below is indicated the previous SARS‐CoV‐2 infection status before vaccination. GMT, Geometric mean titer. Uninfected cancer patients *n* = 67; infected cancer patients *n* = 22; uninfected controls *n* = 15; infected controls *n* = 11.

To assess the response to vaccination, we determined the levels of neutralizing antibodies in plasma at different time points using a pseudovirus‐based neutralization assay against the ancestral strain of SARS‐CoV‐2 (WH1). Several studies have shown that SARS‐CoV‐2 serostatus is one of the major determinants of response [20, 21, 22, 23]. Consistently, we observed significant differences in neutralizing antibody titer between individuals with and without previous SARS‐CoV‐2 infection, (defined by positive PCR in medical records or detectable baseline neutralizing activity prior to vaccination) at baseline (*P* < 0.001), after the first dose (*P* < 0.001) and after the second dose (*P* = 0.031, Fig. 2). In all cases, individuals with the previous infection showed higher levels of neutralization even 15–21 days post‐2^nd^ dose. Significantly, six individuals (6.7%) responded weakly to vaccination (ID_50_ < 500 post‐2^nd^ dose), and one of them generated practically no neutralizing antibodies (ID_50_ = 75 post‐2^nd^ dose); however, these poor responder individuals did not share any clinical and demographic characteristics. When analyzing the neutralizing response 15 day post‐two dose vaccination, we observed significant differences between uninfected patients with cancer compared to the control group (*P* = 0.013). In contrast, this difference was lost when comparing oncology and control individuals with previous infection (*P* = 0.084).

### 3.4 Impact of anticancer therapy on the neutralizing humoral response

**Fig. 3:**
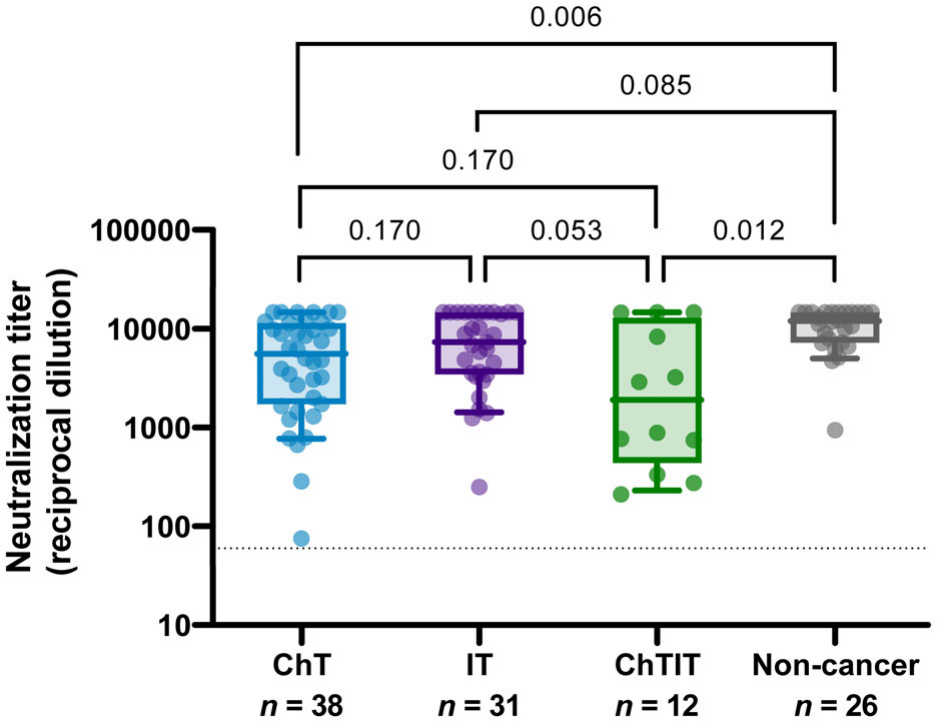
Neutralization titer regarding cancer treatment. Boxes show the median and the interquartile range (25^th^ and 75^th^ percentiles) and whiskers the 10^th^ and 90^th^ percentiles. *P* values correspond to Peto–Peto rank test with Benjamini & Hochberg adjustment. ChT, Chemotherapy; ChTIT, Chemoimmunotherapy; IT, Immunotherapy.

Since the anticancer treatment may influence the immune response to vaccines [24], we analyzed the neutralizing activity post‐2^nd^ dose considering the different groups of active anticancer therapy present in the studied cohort, independently of their previous SARS‐CoV‐2 infection status (Fig. 3). We observed that there was no difference in neutralizing antibody titer between patients on chemotherapy (ID_50_ [GMT] = 4072) and immunotherapy (ID_50_ [GMT] = 5958; *P* = 0.170). The group undergoing chemoimmunotherapy had nearly significantly lower levels (ID_50_ [GMT] = 1906) than those treated with immunotherapy alone (*P* = 0.053), but not with the group of patients treated exclusively with a chemotherapy regimen (*P* = 0.170). Interestingly, the control untreated (non‐cancer) group generated a higher neutralizing antibody titer (ID_50_ [GMT] = 9715); statistical analysis showed significant differences between the untreated group and the chemotherapy group (*P* = 0.006), as well as with the chemoimmunotherapy group (*P* = 0.012). However, there was no statistical difference with patients undergoing immunotherapy alone (*P* = 0.085).

When we exclusively analyzed individuals without previous infection (Fig. S1), we still observed higher antibody titers in control non‐cancer individuals (ID_50_ [GMT] = 9127) compared to cancer patients (global ID_50_ [GMT] = 3605). The results were consistent with the previous analysis, observing differences between non‐cancer and ChT groups (ID50 [GMT] = 3571; *P* = 0.040), although it was not statistically significant between non‐cancer and ChTIT groups (ID50 [GMT] = 1710; *P* = 0.057), probably due to lower statistical power.

No other clinical‐demographic variables, such as sex, age, cancer type, cancer stage, concomitant treatments (oral corticosteroid), analytical parameters such as leukocyte count and NLR, or comorbidities, were significantly associated with the neutralizing humoral response.

## 4 Discussion

Considering the increased risk of complications in cancer patients in case of infections, including SARS‐CoV‐2 infection, preventive measures must be a prime concern. Therefore, cancer patients have been prioritized for vaccinations for seasonal infections such as influenza and now for SARS‐CoV‐2. Many antitumor treatments can cause myelosuppression and immunosuppression, which have been associated with a reduced efficacy of vaccines in patients with the hematologic disease, but not in patients with solid tumors [25, 26, 27]. Since pivotal SARS‐CoV‐2 vaccine studies did not include patients with active anticancer treatment, data coming from real‐world studies are of utmost importance to collect information on both the safety and efficacy of SARS‐CoV‐2 vaccines. Our cohort includes, mostly, patients with advanced lung and breast cancer with a good performance status, a high proportion of smokers, and medium comorbid burden with no detectable statistical differences between therapeutic groups.

In vaccinated individuals, higher levels of neutralizing antibodies have been reported in those with preexisting immunity to SARS‐CoV‐2 compared to naïve individuals, resulting in superior protection against reinfection [20, 28, 29]. Consistent with this, in our oncology patients, a greater magnitude of the neutralizing response was observed in those with the previous infection than uninfected ones. In addition, the neutralizing response was significantly reduced in the uninfected patients on anticancer therapy compared to those uninfected in the non‐cancer control group. These results suggest that a vaccination boost should be recommended in patients receiving anticancer treatment irrespective of the type of therapy, but especially in those without previous SARS‐CoV‐2 infection [30, 31]. Moreover, the emergence of new virus variants of concerns, such as Omicron, reinforces the need for periodical boosts, especially in patients belonging to risk groups such as cancer patients [32, 33]. In our cohort, until the final analysis, 10% of the cohort experienced a SARS‐CoV‐2 infection post‐vaccination. Of these, 67% had mild symptomatology, while 33% experienced severe COVID‐19 requiring hospitalization, with no COVID‐19‐associated deaths. However, cancer mortality after 1 year of vaccination was high (37%), thus, the incidence of breakthorough infections could be underestimated.

Regarding the impact of the different anticancer therapies included in this study on the neutralizing humoral response to the mRNA‐1237 vaccine, we observed significant results when performing statistical analyses considering the entire cohort, but also when only uninfected individuals were analyzed. Our results highlight the impact of different therapies, especially those containing chemotherapeutic agents, in which a lower neutralizing response against SARS‐CoV‐2 was observed. Although this observation is probably associated with the immunosuppressive effect of chemotherapy compared to immunotherapy [34], we failed to identify a clear association between neutralization titers and lymphocyte counts. Therefore, these differences might also be related to a more profound immune dysfunction associated with disease progression [35]. Additionally, cancer patients under treatment with previous SARS‐CoV‐2 infection elicited higher neutralizing antibodies titers compared to uninfected. This might impact SARS‐CoV‐2 reinfections or severity, as it has been reported in healthy populations [36]. However, some limitations of the study prevent us from drawing conclusions and leave open questions, which should be addressed in more detail in larger cohort studies.

Our study identified a small percentage of patients (6.7%) in the treatment group with low neutralization values after the second vaccination dose compared to the non‐cancer control group. These patients would probably be at greater risk of infection, reinfection, and complications in case of infections [37, 38].

In terms of toxicity, there was concern about the vaccination response in patients receiving IT, considering that the over‐activation of the immune system could have led to increased toxicity. Similar to other reports, our results confirm that the AE rate did not increase in this group of patients [16, 39, 40]. Moreover, the AE rate in our cohort is slightly lower than in pivotal studies. The chronic use of pain medication in patients with cancer could explain lower rates of local pain and systemic AE after vaccination.

Our analysis is mainly limited by the reduced sample size, which is insufficiently powered to assess comorbidities as factors that could determine the humoral response, and the lack of consideration of spike‐specific B cells, and T‐cell response that has also been shown to play a central role in preventing complications after reinfections [41, 42]. The main criteria for the identification of infected individuals prior to vaccination were the presence of detectable neutralizing antibodies at baseline; therefore, infected individuals with poor humoral responses or rapid antibody decline could have been misclassified. The fact that the higher proportion of patients in our cohort had lung cancer may limit the generalization of our results to all solid tumor patients. However, the differential immunosuppressors effect may be more important among different therapies than among different tumor origins. This study was carried out when the initial vaccination programs started in March 2021, if the results could apply to novel variants of concern remains unknown.

## 5 Conclusions

In conclusion, this study strengthens the evidence to prioritize patients under cancer treatment for SARS‐CoV‐2 vaccination. Moreover, no additional toxicities have been identified in patients receiving cancer treatment. Since the healthcare facilities' attendance rate will be higher in patients with active anticancer therapy, measures such as facial masking, social distancing and cleaning should also be maintained in oncology facilities.

## Conflict of interest

Outside the submitted work JB is founder and shareholder of AlbaJuna Therapeutics, S.L. BC is founder and shareholder of AlbaJuna Therapeutics, S.L and AELIX Therapeutics, S.L. EF declares advisory role fees from Novartis; travel expenses fees from Lilly, Novartis and Pfizer; and a research funding from Pfizer. MB declares consulting/advisory role fees from Roche, Bristol‐Myers, Boehringer, AstraZeneca, Lilly, and a research funding from Kyowa Kirin. The other authors declare no conflict of interest.

## Author contributions

JB, EF, EP, EB and BC designed and coordinated the study. EP, SM and BT performed and analyzed the neutralization assays. VU, EF and EP performed the statistical analysis. EF, MR, AH, MC, LM, MM and TM selected the patients and coordinated the data. EF, EP, TM and JB drafted the manuscript, and all authors made substantial contributions to the revision of the subsequent versions. All authors approved the submitted version of the manuscript and agreed both to be personally accountable for their own contributions and to answer the questions related to the accuracy or integrity of any part of the work.

### Peer review

The peer review history for this article is available at https://publons.com/publon/10.1002/1878‐0261.13359.

## Supporting information


**Fig. S1.** Neutralization titer regarding cancer treatment in uninfected individuals.

**Table S1.** Treatment regimens.
**Table S2.** Cohort characteristics.
**Table S3.** Adverse effects depending on treatment group.
**Table S4.** Adverse effects depending on previous SARS‐CoV‐2 infection.

## Data Availability

Data are available on request from the authors.

## References

[1] Liang W , Guan W , Chen R , Wang W , Li J , Xu K , et al. Cancer patients in SARS‐CoV‐2 infection: a nationwide analysis in China. Lancet Oncol. 2020;21(3):335–7. 32066541 10.1016/S1470-2045(20)30096-6 PMC7159000

[2] Huang C , Wang Y , Li X , Ren L , Zhao J , Hu Y , et al. Clinical features of patients infected with 2019 novel coronavirus in Wuhan, China. Lancet. 2020;395(10223):497–506. 31986264 10.1016/S0140-6736(20)30183-5 PMC7159299

[3] Khoury E , Nevitt S , Madsen WR , Turtle L , Davies G , Palmieri C . Differences in outcomes and factors associated with mortality among patients with SARS‐CoV‐2 infection and cancer compared with those without cancer: a systematic review and meta‐analysis. JAMA Netw Open. 2022;5(5):e2210880. 35532936 10.1001/jamanetworkopen.2022.10880 PMC9086843

[4] Lee LYW , Cazier J‐B , Starkey T , Briggs SEW , Arnold R , Bisht V , et al. COVID‐19 prevalence and mortality in patients with cancer and the effect of primary tumour subtype and patient demographics: a prospective cohort study. Lancet Oncol. 2020;21(10):1309–16. 32853557 10.1016/S1470-2045(20)30442-3 PMC7444972

[5] Dettorre GM , Dolly S , Loizidou A , Chester J , Jackson A , Mukherjee U , et al. Systemic pro‐inflammatory response identifies patients with cancer with adverse outcomes from SARS‐CoV‐2 infection: the OnCovid inflammatory score. J Immunother Cancer. 2021;9(3):e002277. 33753569 10.1136/jitc-2020-002277 PMC7985977

[6] Gottlieb RL , Vaca CE , Paredes R , Mera J , Webb BJ , Perez G , et al. Early Remdesivir to prevent progression to severe Covid‐19 in outpatients. N Engl J Med. 2022;386(4):305–15. 34937145 10.1056/NEJMoa2116846 PMC8757570

[7] Pinato DJ , Patel M , Scotti L , Colomba E , Dolly S , Loizidou A , et al. Time‐dependent COVID‐19 mortality in patients with cancer: an updated analysis of the OnCovid registry. JAMA Oncol. 2022;8(1):114–22. 34817562 10.1001/jamaoncol.2021.6199 PMC8777559

[8] İlgün AS , Özmen V . The impact of the COVID‐19 pandemic on breast cancer patients. Eur J Breast Health. 2022;18(1):85–90. 35059596 10.4274/ejbh.galenos.2021.2021-11-5 PMC8734528

[9] Drake TM , Riad AM , Fairfield CJ , Egan C , Knight SR , Pius R , et al. Characterisation of in‐hospital complications associated with COVID‐19 using the ISARIC WHO clinical characterisation protocol UK: a prospective, multicentre cohort study. Lancet. 2021;398(10296):223–37. 34274064 10.1016/S0140-6736(21)00799-6 PMC8285118

[10] Baden LR , El Sahly HM , Essink B , Kotloff K , Frey S , Novak R , et al. Efficacy and safety of the mRNA‐1273 SARS‐CoV‐2 vaccine. N Engl J Med. 2021;384(5):403–16. 33378609 10.1056/NEJMoa2035389 PMC7787219

[11] Rousseau B , Loulergue P , Mir O , Krivine A , Kotti S , Viel E , et al. Immunogenicity and safety of the influenza a H1N1v 2009 vaccine in cancer patients treated with cytotoxic chemotherapy and/or targeted therapy: the VACANCE study. Ann Oncol. 2012;23(2):450–7. 21576285 10.1093/annonc/mdr141

[12] Meerveld‐Eggink A , de Weerdt O , van der Velden AMT , Los M , van der Velden AWG , Stouthard JML , et al. Response to influenza virus vaccination during chemotherapy in patients with breast cancer. Ann Oncol. 2011;22(9):2031–5. 21303799 10.1093/annonc/mdq728

[13] Patone M , Mei XW , Handunnetthi L , Dixon S , Zaccardi F , Shankar‐Hari M , et al. Risks of myocarditis, pericarditis, and cardiac arrhythmias associated with COVID‐19 vaccination or SARS‐CoV‐2 infection. Nat Med. 2022;28(2):410–22. 34907393 10.1038/s41591-021-01630-0 PMC8863574

[14] Terán Brage E , Roldán Ruíz J , González Martín J , Oviedo Rodríguez JD , Vidal Tocino R , Rodríguez Diego S , et al. Fulminant myocarditis in a patient with a lung adenocarcinoma after the third dose of modern COVID‐19 vaccine. A case report and literature review. Curr Probl Cancer Case Rep. 2022;6:100153. 35378738 10.1016/j.cpccr.2022.100153 PMC8968161

[15] Kian W , Zemel M , Kestenbaum EH , Rouvinov K , Alguayn W , Levitas D , et al. Safety of the BNT162b2 mRNA COVID‐19 vaccine in oncologic patients undergoing numerous cancer treatment options: a retrospective single‐center study. Medicine (Baltimore). 2022;101(2):e28561. 35029223 10.1097/MD.0000000000028561 PMC8758044

[16] Oosting SF , van der Veldt AAM , GeurtsvanKessel CH , Fehrmann RSN , van Binnendijk RS , Dingemans AC , et al. mRNA‐1273 COVID‐19 vaccination in patients receiving chemotherapy, immunotherapy, or chemoimmunotherapy for solid tumours: a prospective, multicentre, non‐inferiority trial. Lancet Oncol. 2021;22(12):1681–91. 34767759 10.1016/S1470-2045(21)00574-X PMC8577843

[17] Pradenas E , Trinité B , Urrea V , Marfil S , Ávila‐Nieto C , Rodríguez de la Concepción ML , et al. Stable neutralizing antibody levels 6 months after mild and severe COVID‐19 episodes. Med (N Y). 2021;2(3):313–20.e4. 10.1016/j.medj.2021.01.005 PMC7847406 33554155

[18] Pradenas E , Trinité B , Urrea V , Marfil S , Tarrés‐Freixas F , Ortiz R , et al. Clinical course impacts early kinetics, magnitude, and amplitude of SARS‐CoV‐2 neutralizing antibodies beyond 1 year after infection. Cell Rep Med. 2022;3(2):100523. 35233547 10.1016/j.xcrm.2022.100523 PMC8784437

[19] Trinité B , Tarrés‐Freixas F , Rodon J , Pradenas E , Urrea V , Marfil S , et al. SARS‐CoV‐2 infection elicits a rapid neutralizing antibody response that correlates with disease severity. Sci Rep. 2021;11(1):2608. 33510275 10.1038/s41598-021-81862-9 PMC7843981

[20] Trinité B , Pradenas E , Marfil S , Rovirosa C , Urrea V , Tarrés‐Freixas F , et al. Previous SARS‐CoV‐2 infection increases B.1.1.7 cross‐neutralization by vaccinated individuals. Viruses. 2021;13(6):1135. 34204754 10.3390/v13061135 PMC8231627

[21] Manisty C , Otter AD , Treibel TA , McKnight Á , Altmann DM , Brooks T , et al. Antibody response to first BNT162b2 dose in previously SARS‐CoV‐2‐infected individuals. Lancet. 2021;397(10279):1057–8. 33640038 10.1016/S0140-6736(21)00501-8 PMC7972310

[22] Goldberg Y , Mandel M , Bar‐On YM , Bodenheimer O , Freedman LS , Ash N , et al. Protection and waning of natural and hybrid immunity to SARS‐CoV‐2. N Engl J Med. 2022;386:2201–12. 35613036 10.1056/NEJMoa2118946 PMC9165562

[23] Pradenas E , Ubals M , Urrea V , Suñer C , Trinité B , Riveira‐Muñoz E , et al. Virological and clinical determinants of the magnitude of humoral responses to SARS‐CoV‐2 in mild‐symptomatic individuals. Front Immunol. 2022;13:860215. 35572570 10.3389/fimmu.2022.860215 PMC9097229

[24] Emens LA , Middleton G . The interplay of immunotherapy and chemotherapy: harnessing potential synergies. Cancer Immunol Res. 2015;3(5):436–43. 25941355 10.1158/2326-6066.CIR-15-0064 PMC5012642

[25] Blanchette PS , Chung H , Pritchard KI , Earle CC , Campitelli MA , Buchan SA , et al. Influenza vaccine effectiveness among patients with cancer: a population‐based study using health administrative and laboratory testing data from Ontario, Canada. J Clin Oncol. 2019;37(30):2795–804. 31465264 10.1200/JCO.19.00354

[26] Grinshpun A , Rottenberg Y , Ben‐Dov IZ , Djian E , Wolf DG , Kadouri L . Serologic response to COVID‐19 infection and/or vaccine in cancer patients on active treatment. ESMO Open. 2021;6(6):100283. 34634634 10.1016/j.esmoop.2021.100283 PMC8469519

[27] Agbarya A , Sarel I , Ziv‐Baran T , Agranat S , Schwartz O , Shai A , et al. Efficacy of the mRNA‐based BNT162b2 COVID‐19 vaccine in patients with solid malignancies treated with anti‐neoplastic drugs. Cancer (Basel). 2021;13(16):4191. 10.3390/cancers13164191 PMC8391288 34439346

[28] Khoury DS , Cromer D , Reynaldi A , Schlub TE , Wheatley AK , Juno JA , et al. Neutralizing antibody levels are highly predictive of immune protection from symptomatic SARS‐CoV‐2 infection. Nat Med. 2021;27(7):1205–11. 34002089 10.1038/s41591-021-01377-8

[29] Bates TA , McBride SK , Leier HC , Guzman G , Lyski ZL , Schoen D , et al. Vaccination before or after SARS‐CoV‐2 infection leads to robust humoral response and antibodies that effectively neutralize variants. Sci Immunol. 2022;7(68):eabn8014. 35076258 10.1126/sciimmunol.abn8014 PMC8939472

[30] Gobbi F , Buonfrate D , Moro L , Rodari P , Piubelli C , Caldrer S , et al. Antibody response to the BNT162b2 mRNA COVID‐19 vaccine in subjects with prior SARS‐CoV‐2 infection. Viruses. 2021;13(3):422. 33807957 10.3390/v13030422 PMC8001674

[31] Patelli G , Pani A , Amatu A , Scaglione F , Sartore‐Bianchi A . Seroconversion after SARS‐CoV‐2 mRNA booster vaccine in cancer patients. Eur J Cancer. 2022;167:175–6. 35370047 10.1016/j.ejca.2022.02.032 PMC8920783

[32] Fendler A , Shepherd STC , Au L , Wu M , Harvey R , Schmitt AM , et al. Omicron neutralising antibodies after third COVID‐19 vaccine dose in patients with cancer. Lancet. 2022;399(10328):905–7. 35090602 10.1016/S0140-6736(22)00147-7 PMC8789238

[33] Zeng C , Evans JP , Chakravarthy K , Qu P , Reisinger S , Song N‐J , et al. COVID‐19 mRNA booster vaccines elicit strong protection against SARS‐CoV‐2 omicron variant in patients with cancer. Cancer Cell. 2022;40(2):117–9. 34986328 10.1016/j.ccell.2021.12.014 PMC8716174

[34] Kyi C , Postow MA . Immune checkpoint inhibitor combinations in solid tumors: opportunities and challenges. Immunotherapy. 2016;8(7):821–37. 27349981 10.2217/imt-2016-0002 PMC5619130

[35] Braun DA , Street K , Burke KP , Cookmeyer DL , Denize T , Pedersen CB , et al. Progressive immune dysfunction with advancing disease stage in renal cell carcinoma. Cancer Cell. 2021;39(5):632–48.e8. 33711273 10.1016/j.ccell.2021.02.013 PMC8138872

[36] Zollner A , Watschinger C , Rössler A , Farcet MR , Penner A , Böhm V , et al. B and T cell response to SARS‐CoV‐2 vaccination in health care professionals with and without previous COVID‐19. EBioMedicine. 2021;70:103539. 34391087 10.1016/j.ebiom.2021.103539 PMC8358275

[37] Ünsal O , Yazıcı O , Özdemir N , Çubukçu E , Ocak B , Üner A , et al. Clinical and laboratory outcomes of the solid cancer patients reinfected with SARS‐CoV‐2. Future Oncol. 2022;18(5):533–41. 34825831 10.2217/fon-2021-0621 PMC8628862

[38] Borgogna C , De Andrea M , Griffante G , Lai A , Bergna A , Galli M , et al. SARS‐CoV‐2 reinfection in a cancer patient with a defective neutralizing humoral response. J Med Virol. 2021;93(12):6444–6. 34260066 10.1002/jmv.27200 PMC8426853

[39] Waissengrin B , Agbarya A , Safadi E , Padova H , Wolf I . Short‐term safety of the BNT162b2 mRNA COVID‐19 vaccine in patients with cancer treated with immune checkpoint inhibitors. Lancet Oncol. 2021;22(5):581–3. 33812495 10.1016/S1470-2045(21)00155-8 PMC8016402

[40] Cavanna L , Citterio C , Biasini C , Madaro S , Bacchetta N , Lis A , et al. COVID‐19 vaccines in adult cancer patients with solid tumours undergoing active treatment: seropositivity and safety. A prospective observational study in Italy. Eur J Cancer. 2021;157:441–9. 34601285 10.1016/j.ejca.2021.08.035 PMC8410513

[41] Moss P . The T cell immune response against SARS‐CoV‐2. Nat Immunol. 2022;23(2):186–93. 35105982 10.1038/s41590-021-01122-w

[42] Marasco V , Carniti C , Guidetti A , Farina L , Magni M , Miceli R , et al. T‐cell immune response after mRNA SARS‐CoV‐2 vaccines is frequently detected also in the absence of seroconversion in patients with lymphoid malignancies. Br J Haematol. 2022;196(3):548–58. 34649298 10.1111/bjh.17877 PMC8653177

