# Supplementary figures and images for: Impact of chemotherapy and/or immunotherapy on neutralizing antibody response to SARS‐CoV‐2 mRNA‐1237 vaccine in patients with solid tumors
Source: Mol Oncol. 2022 Dec 30;17(4):686–94. doi: 10.1002/1878-0261.13359 (PMC9877816; doi:10.1002/1878-0261.13359)

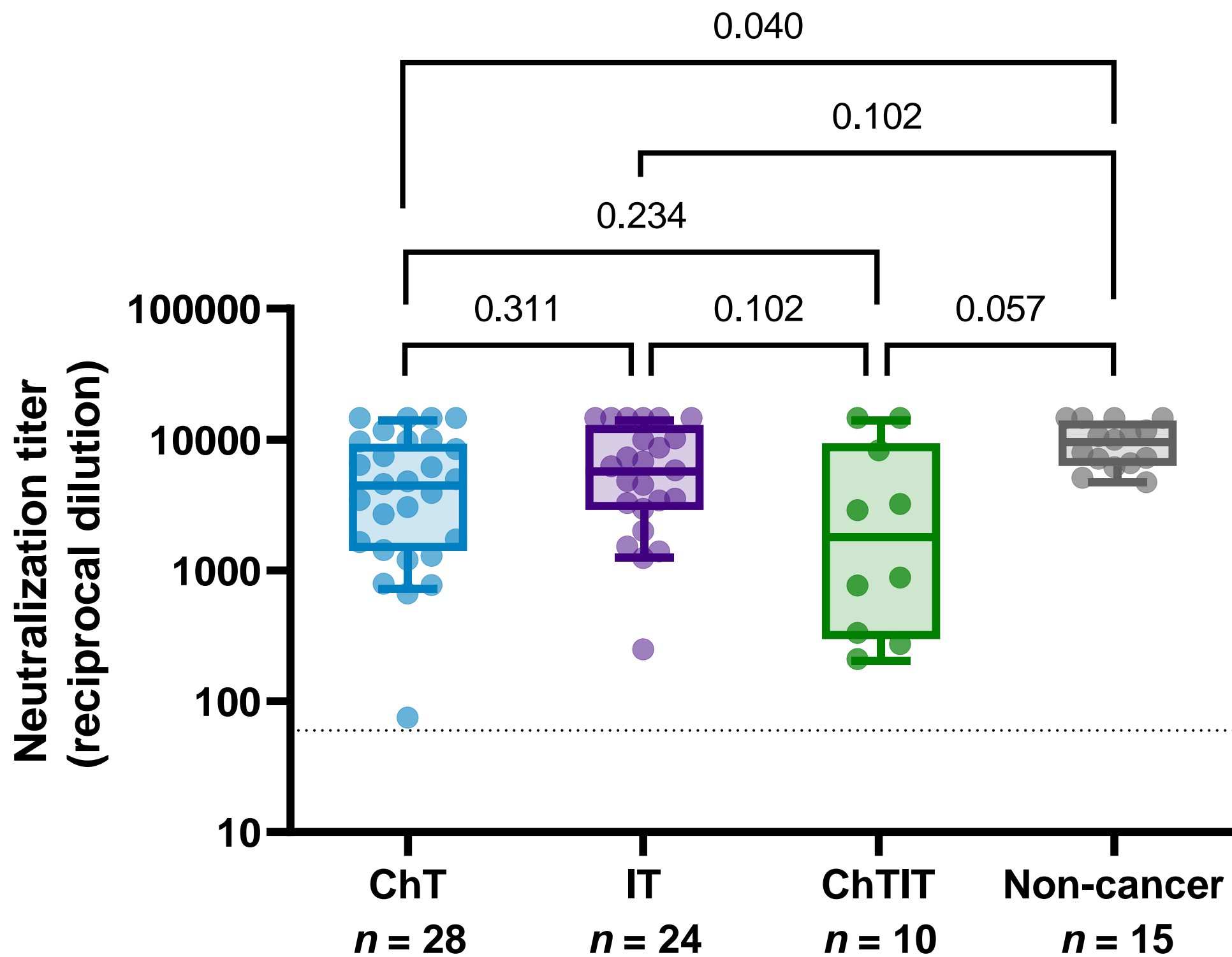

Supplement: Supplementary file 1 — Fig. S1. Neutralization titer regarding cancer treatment in uninfected individuals. [file MOL2-17-686-s002.pdf]
