# Supplementary material for: Impact of chemotherapy and/or immunotherapy on neutralizing antibody response to SARS‐CoV‐2 mRNA‐1237 vaccine in patients with solid tumors
Source: Mol Oncol. 2022 Dec 30;17(4):686–94. doi: 10.1002/1878-0261.13359 (PMC9877816; doi:10.1002/1878-0261.13359)
Supplement: Supplementary file 2 — Table S1. Treatment regimens. Table S2. Cohort characteristics. Table S3. Adverse effects depending on treatment group. Table S4. Adverse effects depending on previous SARS‐CoV‐2 infection. [file MOL2-17-686-s001.rtf]

Treatment	Mechanism of action	n patients	Group	
Platinum chemotherapies combinations	Platinum based ChT	15	ChT	
Taxanes chemotherapies	Anti-microtubule	8	ChT	
Capecitabine	Anti-metabolite	5	ChT	
Pemetrexed	Anti-metabolite	3	ChT	
TDM-1	Anti-microtubule plus anti-HER2	3	ChT	
Bevacizumab + Chemotherapy	Anti-VEGF plus platinum-based ChT	3	ChT	
Vinorelbine	Anti-microtubule	2	ChT
	
Eribulin	Anti-microtubule	1	ChT	
FOLFOX scheme	Platinum-based ChT plus Anti-metabolite	1	ChT	
Pembrolizumab	Anti-PD-1	14	IT	
Nivolumab	Anti-PD-1	9	IT	
Durvalumab	Anti-PD-L1	6	IT	
Atezolizumab	Anti-PD-L1	5	IT	
Pembrolizumab + Chemotherapy	Anti-PD-1	10	ChTIT	
Atezolizumab + Chemotherapy	Anti-PD-L1 plus platinum-based ChT	2	ChTIT	
Avelumab + Chemotherapy	Anti-PD-L1	1	ChTIT	
Durvalumab + Chemotherapy	Anti-PD-L1	1	ChTIT	

Supplementary Table 1. Treatment regimens. The number of patients in each treatment and the group of treatment considered in the study. ChT: chemotherapy; IT: immunotherapy; ChTIT: chemoimmunotherapy; n: number of patients


Characteristic	ChT, n = 41	IT, n = 34	ChTIT, n = 14	p-value1	
Baseline comorbidities, n (%)					
Diabetes	9 (22%)	10 (29%)	6 (43%)	0.315	
Hypertension	15 (37%)	14 (41%)	8 (57%)	0.423	
Cardiac disease	6 (15%)	9 (26%)	5 (36%)	0.204	
Respiratory disease	11 (27%)	16 (47%)	6 (43%)	0.177	
Renal failure	1 (2.4%)	4 (12%)	2 (14%)	0.183	
Concomitant treatment, n (%)					
Oral corticosteroids 	3 (7.3%)	3 (8.8%)	3 (21,4%)	0.303	
Baseline tobacco exposure, n (%)				<0.001	
Never smokers	20 (49%)	1 (2.9%)	3 (21%)		
Former smokers	14 (34%)	20 (59%)	7 (50%)		
Current smokers	7 (17%)	13 (38%)	4 (29%)		
Baseline analytical results, median [IQR]  					
Leucocytes (x109cells/liter) 	5.8 [4.4-7.3]	7.6 [5.9-9.2]	6.2 [4.7-9.4]	0.018	
Neutrophils (x109cells/liter)	3.3 [2.3-4.8]	4.9 [3.7-6.3]	4.3 [2.5-6.3]	0.016	
Lymphocytes (x109cells/liter)	1.5 [1.1-2.0]	1.4 [0.9-1.9]	1.4 [1.2-1.9]	0.801	
NLR 	2.0 [1.4-3.4]	3.0 [2.0-6.3]	2.7 [1.8-4.2]	0.019	
Hemoglobin (grams/deciliter) 	11.7 [10.9-12.9]	13.2 [12.2-14.3]	11.9 [11.3-13.1]	0.001	
Platelets (x109cells/liter)	209 [167-268]	235 [192-295]	262 [147-397]	0.311	
Pre-2nd dose analytical results, median [IQR]					
Leucocytes (x109cells/liter)	5.7 [4.3-7.6]	7.3 [6.0-9.0]	6.0 [4.8-8.5]	0.042	
Neutrophils (x109cells/liter)	3.4 [2.2-5.3]	4.6 [3.7-6.4]	3.6 [2.3-4.9]	0.070	
Lymphocytes (x109cells/liter)	1.4 [1.1-1.9]	1.7 [0.9-2.2]	1.6 [1.1-2.0]	0.623	
NLR	2.1 [1.6-4.1]	3.1 [1.8-5.8]	2.0 [1.7-2.7]	0.321	
Hemoglobin (grams/deciliter)	11.6 [10.7-12.6]	13.4 [12.8-14.3]	11.6 [10.2-12.7]	<0.001	
Platelets (x109cells/liter)	213 [165-283]	216 [187-269]	236 [197-370]	0.326	
					
Current status, n (%)				0.188	
Death	17 (41.4%)	10 (29.4%)	6 (42.9%)		
Alive	24 (58.6%)	24 (70.6%)	8 (57.1%)		
SARS-CoV-2 infection after vaccination, n (%)				0.567	
	5 (12.2%)	2 (5.9%)	2 (14.3%)		

1Pearson's Chi-squared test; Fisher's exact test; Kruskal-Wallis rank sum test
Supplementary Table 2. Cohort characteristics. Baseline comorbidities, concomitant treatment and tobacco exposure, blood analysis values before first and second vaccine dose administration, and status of the cohort at the time of analysis. ChT: chemotherapy; IT: immunotherapy; ChTIT: chemoimmunotherapy; n: number of patients NLR: Neutrophil/Lymphocyte ratio
Adverse effects, n (%)	ChT, n = 41	IT, n = 34	ChTIT, n = 14	p-value1	
Local adverse effects	27 (65.9%)	23 (67.6%)	8 (57.1%)		
Pain	25 (61.0%)	17 (50.0%)	7 (50.0%)		
Warmness	6 (14.6%)	4 (11.8%)	2 (14.3%)		
Redness	3 (7.3%)	6 (17.6%)	2 (14.3%)		
				0.891	
Systemic adverse effects 	18 (43.9%)	17 (50.0%)	6 (42.9%)		
Fatigue 	10 (24.4%)	12 (35.3%)	3 (21.4%)		
Fever 	9 (22.0%)	10 (29.4%)	4 (28.6%)		
Muscle and joint pain	5 (12.2%)	5 (14.7%)	2 (14.3%)		
Headache	4 (9.8%)	8 (23.5%)	1 (7.1%)		
				0.977	

1Pearson's Chi-squared test to assess differences in distribution between groups
Supplementary Table 3. Adverse effects depending on treatment group. ChT: chemotherapy; IT: immunotherapy; ChTIT: chemoimmunotherapy; n: number of patients
			


Characteristic	Previous SARS-CoV-2 infection, n = 22	No previous SARS-CoV-2 infection, n = 67	p-value1	
Local adverse effects	12 (54.5%)	46 (68.7%)		
Pain	8 (36.4%)	41 (61.2%)		
Warmness	5 (22.7%)	7 (10.4%)		
Redness	1 (4.5%)	4 (6.0%)		
			0.290	
Systemic adverse effects 	10 (45.5%)	31 (46.3%)		
Fatigue 	6 (27.3%)	19 (28.4%)		
Fever 	6 (27.3%)	17 (25.4%)		
Muscle and joint pain	3 (13.6%)	9 (13.4%)		
Headache	1 (4.5%)	7 (10.4)		
			0.958	

1Pearson's Chi-squared test to assess differences in distribution between groups
Supplementary Table 4. Adverse effects depending on previous SARS-CoV-2 infection.
